# Atypical Positional Vertigo: Definition, Causes, and Mechanisms

**DOI:** 10.3390/audiolres12020018

**Published:** 2022-03-14

**Authors:** Sergio Carmona, Guillermo Javier Zalazar, Martin Fernández, Gabriela Grinstein, João Lemos

**Affiliations:** 1 Fundación San Lucas Para la Neurociencia, Rosario 2000, Argentina; guille.zalazar87@gmail.com (G.J.Z.); fmartingabriel@gmail.com (M.F.); 2 Instituto de Neurociencias de Buenos Aires INEBA, Buenos Aires 1192, Argentina; gabrielagrin@gmail.com; 3 Department of Neurology, Hospital Central Dr. Ramón Carrillo, San Luis 5700, Argentina; 4 Department of Neurology, Coimbra University Hospital Centre, 3004-561 Coimbra, Portugal; merrin72@hotmail.com

**Keywords:** APV (atypical positional vertigo), heavy cupula, light cupula, vestibular migraine, apogeotropic PV, vertigo in childhood

## Abstract

Paroxysmal positional vertigo is a frequent cause for consultation. When approaching these patients, we try to differentiate central from peripheral causes, but sometimes we find manifestations that generate diagnostic doubts. In this review, we address atypical paroxysmal positional vertigo, reviewing the literature on the subject and giving a provisional definition of atypical positional vertigo as well as outlining its causes and pathophysiological mechanisms.

## 1. Introduction

Benign paroxysmal positional vertigo is the most frequent cause of vertigo [1]. As its name indicates, it is characterized by vertigo episodes of sudden onset and end, triggered by changes in head’s position with regard to gravity. It is located in the labyrinth, and its cause is mechanical [2]. However, this is an etiologic diagnosis, reached after questioning and examining the patient.

Based on what patients report, the duration of symptoms lasts seconds; however, many overestimate the duration of the vertiginous sensation. The trigger effect of positional changes is a key issue to be addressed [2]. A great variability of autonomic symptoms, including nausea and vomiting, can accompany BPPV. Gait instability, headache, and additional neurologic complaints are potential red flags in the differential diagnosis [1]. With a defined position trigger effect, it is the neurologist’s job to perform an examination to confirm the diagnosis of paroxysmal positional vertigo (PPV), and by virtue of the vertigo duration and nystagmus characteristics, to determine lesion localization (peripheral versus central) and to design a management plan.

Therefore, we may define PPV as a condition characterized by sudden-onset and -end vertigo episodes, triggered by changes in the head’s position with regard to gravity.

If, after questioning, the diagnosis is PPV, there is an attempt to find the cause of these symptoms by performing a physical examination and, in certain cases, supplementary tests. The causes may be divided in two main groups: Peripheral, as in the case of Benign Paroxysmal Positional Vertigo (BPPV), in its typical and atypical forms [3], and positional alcohol nystagmus [4]; and Central (Central Paroxysmal Positional Vertigo, or CPPV), due to multiple causes, for instance, vascular, demyelinating, degenerative, or nutritional [5].

In 1999, Büttner et al. published criteria to help differentiate between BPPV and CPPV [6]. Soto-Varela et al. proposed an update of the previous differentiation criteria [7], as follows:

- PPV associated to neurological disorder signs and symptoms;
- Nystagmus without vertigo in positional maneuvers;
- Atypical direction nystagmus, especially downbeating nystagmus;
- Nystagmus that changes direction during the positional test;
- Poor response to repositioning maneuvers;
- Recurrence on more than three occasions, confirmed by positional tests.

These elements are usually useful to differentiate the aforementioned clinical pictures when they are typical (for a full description of the diagnostic criteria of the different BPPV variants, we recommend reading the diagnostic criteria published by the Bárány Society [1]). In practice, clinical pictures that do not fall within the usual description are common. For instance, in recent years some “atypical” semiological findings have been described for BPPV, as may be the case for direction-fixed positional nystagmus [8] and downbeat nystagmus due to posterior canal compromise (apogeotropic posterior canal nystagmus) [9,10,11].

Below, we provide a brief review of some of the PPV causes that, due to their characteristics, may result in diagnostic doubts. We leave aside the description of typical BPPV pictures (see [1]), but provide a definition of atypical PPV and describe the variants found in and supported by the literature.

## 2. Atypical Positional (APV)

In most patients, BPPV presents with a typical description and physical examination, with no diagnostic difficulties. However, in some cases, there are hard-to-explain findings, which may result in diagnostic error.

**Figure 1:**
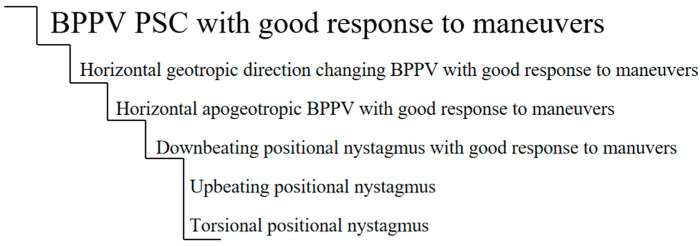
Ladder of atypicality.

If we were to use a ladder to plot the degree of atypicality, we may find what is shown in Figure 1. This chart is a proposed ladder and is not intended to be definitive. At the top, we have the most frequent BPPV, one with posterior canal compromise and with an excellent response to maneuvers. As we go down the ladder, we find less frequent forms, which may overlap with positional vertigo of central causes.

In recent years, many cases and series of cases have been published [12,13,14] of patients with a clinical picture of positional vertigo, who were initially diagnosed as BPPV but whose final diagnosis was of CPPV or vestibular neuritis; these cases match what is represented in the ladder.

Other clinical pictures of atypical nystagmus have been described for BPPV, which were explained by the presence of otoconial particles in different parts of the canals. The characteristic they shared was that, in their evolution, after the maneuvers were performed, the typical findings of the affected canal BPPV appeared and were solved with repositioning maneuvers [8,9,10,11,15]. These clinical pictures would be atypical BPPVs.

Based on the findings of the aforementioned papers, we propose the following elements for a positional vertigo to be considered atypical:

- APV based on epidemiology: pediatric age, except post-HT [14,16];
- APV based on direction of the nystagmus: purely torsional (purely vertical) change in the direction of the nystagmus [5];
- APV based on duration: no latency, excessive duration [6,7];
- APV based on therapeutic test: lack of response to maneuvers [5,6,7,12,13,14];
- APV based on evolution: persistence of signs throughout time [5,6,7,13].

Below we present some atypical BPPVs that have been described recently.

### 2.1. Direction-Fixed Positional Nystagmus

Calífano et al. [8] described the lateral canal BPPV as a condition characterized by the presence of direction-fixed positional nystagmus (for instance, apogeotropic nystagmus with roll test to the left and geotropic with roll test to the right). Diagnosis is confirmed by the subsequent transformation to a typical lateral canal BPPV with direction-changing nystagmus. The mechanism proposed includes the presence of particles of different sizes and densities in the same canal, with the larger ones trapped in the narrow area of the canal.

### 2.2. Posterior Canal Apogeotropic BPPV Variant

**Figure 2:**
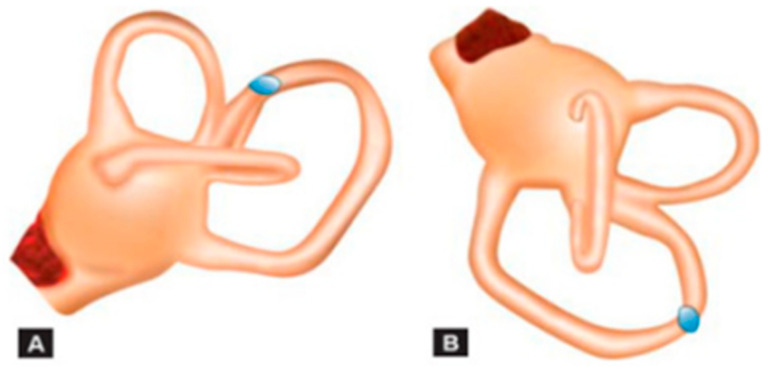
The debris inside the posterior semicircular canal (PSC) close to the common crus (**A**), gravitating toward the PSC ampullary arm (**B**).

In 2012, Vanucchi et al. [10] described a series of patient cases that, when examined, presented a positional downbeat nystagmus, which then evolved into a posterior canal BPPV caused by cupulolitiasis. Since then, many reports have noted patients with similar clinical pictures [9,11,17]. This clinical picture is suspected when the anterior canal repositioning maneuvers do not bring relief, or when a nystagmus typical of a posterior canal compromise appears in subsequent tests. It is suspected that the particles are near the common crus, and that, when the position of the Dix Hallpike maneuver is performed, they create a centripetal current, which inhibits the posterior canal and causes a downbeat nystagmus, with a torsional component that may not always be present [11] (Figure 2). Another proposed mechanism is the presence of posterior canal cupulolitiasis [3].

### 2.3. Multi-Canal BPPV

We must differentiate between the more common “canal change” phenomenon, which may be seen when the repositioning maneuvers are not properly performed for the compromised canal, and the more uncommon simultaneous compromise of both canals, which may be observed in cases secondary to head trauma. In these cases, a positional nystagmus with horizontal and torsional components of a magnitude similar to that evoked by the Dix-Hallpike maneuver can be observed; also, a positional upbeat vertical and torsional nystagmus can be obtained with the Dix-Hallpike maneuver to one side, and a positional horizontal direction-changing nystagmus can be obtained with the roll test. The reason for this is that a compromise of the lateral and posterior canal on the same side is the most frequent situation. Compromising of more canals is not frequent [15].

### 2.4. Sitting-Up Vertigo

A clinical picture of positional vertigo with upbeat nystagmus has been described, which appears upon the patient sitting up. Scocco et al. [18] propose that this is a variant of the posterior canal BPPV, where the presence of a narrowing in the canal would prevent the particles near the cupula from moving when the patient adopts the Dix-Hallpike position, but which, upon sitting up, will first produce an ampullopetal flow that does not translate into nystagmus and then a more intense ampullofugal flow, which would translate into an upbeat nystagmus with a torsional component.

### 2.5. Spontaneous Nystagmus

Persistent spontaneous nystagmus has been described in lateral and posterior canal BPPV [19,20,21,22]. It is not influenced by gravity and does not change direction with maneuvers: this differentiates it from pseudospontaneous nystagmus, which changes with movements in the sagittal and horizontal planes. Spontaneous nystagmus is attributed to the presence of particles stuck within a narrow segment of the semicircular canal (canalithic jam or functional plugging), causing positive or negative endolymphatic pressure and persistent deflection of the cupula. It usually appears after repositioning maneuvers and more frequently affects the lateral canal. If it does not appear after therapeutic procedures, it is difficult to differentiate from vestibular neuritis nystagmus, because functional plugging of the canal can also lead to reversible caloric paresis [19,20].

## 3. Cupula-Endolymph Density Alteration

These alterations, though not totally defined, should be taken into account as they

1. are the cause of APV, and
2. do not respond to repositioning maneuvers.

### 3.1. Heavy Cupula

A heavy cupula is characterized by the presence of persistent (lasting longer than a minute) apogeotropic positional nystagmus with cephalic changes and of a null point. Some authors have proposed that the density of the cupula would increase as regards the endolymphatic density, thus producing an ampullofugal deflection that would facilitate the persistence of the position-changing apogeotropic nystagmus, depending on the cephalic position. Under normal conditions, the semicircular canals do not depend on gravity, taking into account that the cupula and the endolymph have the same density and therefore the same gravity. However, if the density of the cupula becomes heavier or lighter as compared to that of the endolymph, its deflection due to the presence of otolith remains (debris) alters its gravitational sensitivity. Hiruma et al. hypothesized that a heavy cupula would actually be more of an otoconial phenomenon than a gravitational change and set forth the possibility of a phenomenon in which particles float (“buoyancy”) in the horizontal canal, contrary to what happens in a light cupula, in which there would be an increase of endolymphatic density. In their 2011 paper, they mentioned that patients with a heavy cupula diagnosis responded to repositioning maneuvers, while light cupula patients did not [23,24].

### 3.2. Light Cupula Syndrome

The light cupula syndrome (LCS) is quite uncommon, but it should be taken into account as it can resemble a horizontal canal BPPV. Patients with LCS usually present with positional vertigo and a constant sensation of imbalance. Nystagmus lasts longer than a minute, is horizontal, geotropic, and direction-changing with head roll. It has no latency or tiredness, showing a constant slow phase velocity that does not fatigue, as is seen in positional alcohol nystagmus phase 1. In the supine position, there is a null point when rotating the head 20 or 30 degrees to the affected side, at which point the nystagmus subsides.

The term light cupula was coined by Shigeno in 2002, as it is believed that a direction-changing persistent nystagmus with head rotations is the result of an anti-gravitational deviation of the cupula in the lateral semicircular canal [25].

When the cupula is light, it becomes gravity sensitive, and therefore, when the head is rotated to the affected side, the cupula will be persistently deflected. When the patient is in a sitting position, a spontaneous nystagmus may observed, which will stop when the head is tilted approximately 30 degrees to the front, as this puts the lateral canal in a position parallel to the horizontal plane. In some patients, the light cupula syndrome may be accompanied by unilateral hearing loss, which suggests that there is a concomitant labyrinth alteration. If the nystagmus is apogeotropic, it could be caused by an increase of endolymphatic density. Patients with LCS are refractory to the attempts to reposition the particles. The course of recovery is usually slow, and it takes some days or weeks [26].

Despite the fact that positional vertigo and nystagmus caused by light cupula are similar to BPPV, it has not yet been determined if they are a variant of BPPV. Their pathogenesis is still unknown, and they are generally considered pathologic vestibular phenomena. There are many theories that explain why the cupula becomes lighter than the endolymph but only in the lateral canal, including the following: light debris attached to the cupula; a reduced cupula density as compared to normal endolymphatic density due to an altered homeostasis of sulphated proteoglycans, which are synthesized in the cupula; an increase in endolymphatic density due to chemical changes and a difference between perilymphatic and endolymphatic densities. Light cupula is still a mystery. The nystagmus it presents is similar to that of phase 1 positional alcohol nystagmus, in which the cupula is relatively lighter than the endolymph, as alcohol, which is less dense than water, enters the cupula quicker than the endolymph [27].

The null point is the most important characteristic in the diagnosis of light cupula [28]. The absence of any sign of alteration of central origin must also be considered, as this is an ear concomitant pathology (Ménière, Ramsay Hunt, Labyrinthitis).

The relatively specific change in cupula and endolymph density is dynamic. Light cupula might not be an independent pathology but a pathological condition or stage of an inner ear pathology [29].

To date, there is no effective treatment for light cupula. The existing canalicular repositioning maneuvers do not solve the problem, and more thorough research is needed to find a specific treatment.

## 4. Apogeotropic and Geotropic Horizontal Nystagmus of Central Cause

It is a well-known fact that the cerebellar nodulus/uvula integrates otolith signals for the translational vestibulo-reflex [30], and though it has always been said that pure horizontal nystagmus, without latency and of long duration, is central, the mechanism has been included in recent publications [12,31] where an abnormal perception causes this form of nystagmus: “If the bias is toward the nose, when the head is turned to the side while supine, there will be sustained, unwanted, horizontal positional nystagmus (apogeotropic type of central positional nystagmus) because of an inappropriate feedback signal indicating that the head is rotating when it is not”.

There is evidence that shows that the apogeotropic forms are caused by lesions in the nodulus, and that the geotropic ones would be caused by a compromise of the floculus [32].

## 5. Vestibular Paroxysmia

Its clinical picture is characterized by brief vertigo attacks, which usually last less than a minute and which might occur many times a day. These episodes usually occur as a result of some cephalic movements and are accompanied by hyperacusis and/or tinnitus [33].

We studied 38 patients with a Vestibular Paroxysmia diagnosis (unpublished data) who sought consultation due to spontaneous vertigo. The average age was 59 years.

Of the 38 patients, 20 (52.6%) were women and 18 (47.4%) were men.

Of the 38 patients, 19 referred positional vertigo, which represents 50%.

In addition, 47.3% (18 patients) presented positional nystagmus in the physical examination. In most of these cases, the characteristic was vertical (13 out of 18).

Hyperventilation was positive in 28.9% (11 patients).

## 6. Vestibular Migraine

One of the types of vertigo that, according to the Bárány Society, a patient may present during a vestibular migraine attack is positional vertigo [34].

Approximately 65% of patients present positional vertigo (1 min to days, plus spontaneous vertigo, migraine symptoms, tinnitus, oscillopsia, feeling of auditory fullness, and/or subjective hearing loss) [35,36,37,38].

Central positional nystagmus is present in up to 100% of the attacks, with or without gait ataxia, which is present in 90% of the attacks [35,36,37,38].

Positional nystagmus has a variable pattern: persistent fixed-direction horizontal nystagmus, apogeotropic, downbeating, upbeating, and torsional nystagmus. Interictal may persist after a mild positional nystagmus in the dark [35,36,37,38].

In our database (unpublished data), where a total of 45 patients with a diagnosis of vestibular migraine and with an average age of 56.3 years were analyzed, 13 consulted due to positional vertigo; out of these patients, the following presented positional nystagmus of variable characteristics: eight had down-beat nystagmus, two had horizontal to the left, and two had vertical with a rotational component.

## 7. Inferior Vestibular Neuritis

Though it is not very common, a clinical picture of vestibular neuritis may produce postural symptoms. We described [12] a patient with compromising of the posterior canal in the context of an inferior vestibular neuritis, who presented paroxysmal positional vertigo when the Dix-Hallpike maneuver was performed to the left, which resulted in a paroxysmal downbeat nystagmus. The vHIT show a gain reduction in the left posterior semicircular canal with corrective saccades, compatible with a clinical picture of inferior vestibular neuritis. A brain MRI was normal, and there was no response to repositioning maneuvers.

## 8. Proposed Definition for APV and Atypical BPPV

We consider that APV is the positional vertigo clinical picture, which

- Is accompanied by neurological disease signs/symptoms (this does not apply to posterior canal BPPV: in many instances, posterior canal BPPV occurs in patients with CNS disorders; it is unrelated to these and improves with an Epley maneuver [39]);
- Appears during childhood, except post-HT;
- Presents a purely direction-changing torsional nystagmus (purely vertical);
- Has no latency;
- Is of excessive duration;
- Does not respond to the maneuvers;
- Presents signs that persist throughout time.

In these cases, we must keep in mind that this could possibly be a clinical picture of central origin.

We consider that a BPPV is atypical when

- The nystagmus does not fall into the classical description for the affected canal;
- During its evolution, the typical signs of the suspected canal being affected appear;
- It responds to repositioning maneuvers;
- Central causes have been ruled out.

## 9. Central Positional Nystagmus and Vertigo

We propose the following classification of central positional nystagmus (Table 1).

## 10. Proposed Definition for APV

*DEFINITIVE APV* means that no maneuver should be applied and that other different diagnoses (migraine, central causes) should be considered and, if applicable, neuroimages should be indicated.

- Purely vertical upbeat positional nystagmus
- Purely torsional positional nystagmus
- Severe truncal ataxia [40]

*PROBABLE APV* means that maneuvers should be performed and evolution should be followed up.

- Though it does not fall into the classical description for the affected canal, the maneuvers result in changes and/or resolution
- Geotropic or apogeotropic positional nystagmus that does not respond to the maneuvers, disappears in time, and in which central causes are ruled out

In Table 2 and Table 3, we enumerate the causes of definite APV and possible APV.

## Figures and Tables

**Table 1:** Classification of central positional nystagmus based on [5].

Classification	Characteristics	Features
Structural Paroxysmal CPN	Frequently multiplanar and aligned Can be mixed	Paroxysmal downbeat nystagmus during straight head hanging and Dix-Hallpike maneuver Paroxysmal upbeat nystagmus when resuming upright position Paroxysmal apogeotropic nystagmus upon Pagnini-McClure Maneuver
		Paroxysmal upbeat nystagmus during straight head hanging and/or Dix-Hallpike maneuver Paroxysmal geotropic nystagmus upon Pagnini-McClure Maneuver
Structural Persistent CPN		Apogeotropic nystagmus during Pagnini-McClure Maneuver Geotropic nystagmus upon Pagnini-McClure Maneuver Downbeat nystagmus during straight head hanging and/or Dix-Hallpike maneuver
Structural Paroxysmal and Persistent CPN	Rarely, nystagmus plane is not aligned	Oblique, torsional, upbeat, or horizontal nystagmus during Dix-Hallpike maneuver Upbeat ortorsional nystagmus during Pagnini-McClure Maneuver Torsional and horizonto-rotatory nystagmus during straight head hanging Torsional nystagmus when sitting up

**Table 2:** Definitive APV causes.

Causes	Nystagmus
Cerebellar lesions	
Flocculus/paraflocculus	Positional downbeating nystagmus
Nodulus	Horizontal direction-changing apogeotropic nystagmus/Positional downbeating nystagmus
Flocculus	Horizontal direction-changing geotropic nystagmus
Vestibular migraine	Variable, mainly central forms
Vestibular paroxysmia	Variable
Tumor in VIII nerve	Horizontal direction-changind nystagmus

**Table 3:** Probable APV causes.

Causes	Nystagmus
Heavy cupula	Horizontal direction-changing apogeotropic nystagmus
Light cupula	Horizontal direction-changing geotropic nystagmus
Sitting up vertigo	Upbeating nystagmus when the patient is sitting up
Apogeotropic variant of posterior semicircular canal	Downbeating positional paroxysmal nystagmus
Vestibular neuritis with differential compromise of vertical canal	Downbeating nystagmus (vestibular inferior neuritis) Upbeating nystagmus (anterior canal neuritis, hypothetical)

## References

[1] Von Brevern M., Bertholon P., Brandt T., Fife T., Imai T., Nuti D., Newman-Toker D. (2017). Benign paroxysmal positional vertigo: Diagnostic criteria Consensus document of the Committee for the Classification of Vestibular Disorders of the Bárány Society. Acta Otorrinolaringol. Esp..

[2] Asprella Libonati G. (2012). Benign Paroxysmal Positional Vertigo and Positional Vertigo Variants. Otorhinolaryngol. Clin. Int. J..

[3] Büki B., Mandalà M., Nuti D. (2014). Typical and atypical benign paroxysmal positional vertigo: Literature review and new theoretical considerations. J. Vestib. Res..

[4] Money K.E., Johnson W.H., Corlett B.M.A. (1965). Role of semicircular canals in positional alcohol nystagmus. Am. J. Physiol..

[5] Lemos J., Strupp M. (2021). Central positional nystagmus: An update. J. Neurol..

[6] Büttner U., Helmchen C., Brandt T. (1999). Diagnostic criteria for central versus peripheral positioning nystagmus and vertigo: A review. Acta Otolaryngol..

[7] Soto-Varela A., Rossi-Izquierdo M., Sánchez-Sellero I., Santos-Pérez S. (2013). Revised criteria for suspicion of non-benign positional vertigo. QJM.

[8] Califano L., Vassallo A., Melillo M.G., Mazzone S., Salafia F. (2013). Direction-fixed paroxysmal nystagmus lateral canal benign paroxysmal positioning vertigo (BPPV): Another form of lateral canalolithiasis. Acta Otorhinolaryngol. Ital..

[9] Cambi J., Astore S., Mandalà M., Trabalzini F., Nuti D. (2013). Natural course of positional down-beating nystagmus of peripheral origin. J. Neurol..

[10] Vannucchi P., Pecci R., Giannoni B. (2012). Posterior semicircular canal benign paroxysmal positional vertigo presenting with torsional downbeating nystagmus: An apogeotropic variant. Int. J. Otolaryngol..

[11] Carmona S., Zalazar G., Weisnchelbaum R., Grinstein G., Breinbauer H., Asprella Libonati G. (2017). Downbeating Nystagmus in Benign Paroxysmal Positional Vertigo: An Apogeotropic Variant of Posterior Semicircular Canal. Curr. Opin. Neurol. Sci..

[12] Carmona S., Grinstein G., Weinschelbaum R., Zalazar G. (2018). Topodiagnosis of the Inner Ear: Illustrative Clinical Cases. Ann. Otolaryngol. Rhinol..

[13] Carmona S., Salazar R., Zalazar G. (2017). Atypical Benign Paroxysmal Positional Vertigo in a Case of Acoustic Neuroma. J. Otolaryngol. ENT Res..

[14] Sergio C., Gabriela G., Romina W., Guillermo Z. (2018). Benign Paroxysmal Positional Vertigo: Differential Diagnosis in Children. Biomed. J. Sci. Technol. Res..

[15] Bertholon P., Chelikh L., Tringali S., Timoshenko A., Martin C. (2005). Combined horizontal and posterior canal benign paroxysmal positional vertigo in three patients with head trauma. Ann. Otol. Rhinol. Laryngol..

[16] Sommerfleck P.A., González Macchi M.E., Weinschelbaum R., De Bagge M.D., Bernáldez P., Carmona S. (2016). Balance disorders in childhood: Main etiologies according to age. Usefulness of the video head impulse test. Int. J. Pediatr. Otorhinolaryngol..

[17] Castellucci A., Malara P., Martellucci S., Botti C., Delmonte S., Quaglieri S., Rebecchi E., Armato E., Ralli M., Manfrin M.L. (2020). Feasibility of Using the Video-Head Impulse Test to Detect the Involved Canal in Benign Paroxysmal Positional Vertigo Presenting with Positional Downbeat Nystagmus. Front. Neurol..

[18] Scocco D.H., García I.E., Barreiro M.A. (2019). Sitting Up Vertigo. Proposed Variant of Posterior Canal Benign Paroxysmal Positional Vertigo. Otol. Neurotol..

[19] Epley J.M. (1995). Positional vertigo related to semicircular canalithiasis. Otolaryngol. Head Neck Surg..

[20] Von Brevern M., Clarke A.H., Lempert T. (2001). Continuous vertigo and spontaneous nystagmus due to canalolithiasis of the horizontal canal. Neurology.

[21] Castellucci A., Malara P., Brandolini C., Del Vecchio V., Giordano D., Ghidini A., Ferri G.G., Pirodda A. (2019). Isolated horizontal canal hypofunction differentiating a canalith jam from an acute peripheral vestibular loss. Am. J. Otolaryngol..

[22] Castellucci A., Malara P., Martellucci S., Delmonte S., Ghidini A. (2020). Fluctuating posterior canal function in benign paroxysmal positional vertigo depending on how and where otoconia are disposed. Otol. Neurotol..

[23] Lagos A.E., Ramos P.H., Aracena-Carmona K., Novoa I. (2021). Conversion from geotropic to apogeotropic direction changing positional nystagmus resulting in heavy cupula positional vertigo: Case report. Braz. J. Otorhinolaryngol..

[24] Hiruma K., Numata T., Mitsuhashi T., Tomemori T., Watanabe R., Okamoto Y. (2011). Two types of direction-changing positional nystagmus with neutral points. Auris Nasus Larynx.

[25] Shigeno K., Oku R., Takahashi H., Kumagami H., Nakashima S. (2001). Static direction-changing horizontal positional nystagmus of peripheral origin. J. Vestib. Res..

[26] Kerber K.A. (2021). Episodic Positional Dizziness. Contin. Lifelong Learn. Neurol..

[27] Nuti D., Zee D.S., Mandalà M. (2020). Benign Paroxysmal Positional Vertigo: What We Do and Do Not Know. Semin. Neurol..

[28] Tang X., Huang Q., Chen L., Liu P., Feng T., Ou Y., Zheng Y. (2019). Clinical Findings in Patients with Persistent Positional Nystagmus: The Designation of “Heavy and Light Cupula”. Front. Neurol..

[29] Zhang S.L., Tian E., Xu W.C., Zhu Y.T., Kong W.J. (2020). Light Cupula: To Be or Not to Be?. Curr. Med. Sci..

[30] Walker M.F., Tian J., Shan X., Tamargo R.J., Ying H., Zee D.S. (2010). The cerebellar nodulus/uvula integrates otolith signals for the translational vestibulo-ocular reflex. PLoS ONE.

[31] Choi J.Y., Kim J.H., Kim H.J., Glasauer S., Kim J.S. (2015). Central paroxysmal positional nystagmus: Characteristics and possible mechanisms. Neurology.

[32] Takemori S., Cohen B. (1974). Loss of visual suppression of vestibular nystagmus after flocculus lesions. Brain Res..

[33] Strupp M., Lopez-Escamez J.A., Kim J.S., Straumann D., Jen J.C., Carey J., Bisdorff A., Brandt T. (2016). Vestibular paroxysmia: Diagnostic criteria. J. Vestib. Res..

[34] Lempert T., Olesen J., Furman J., Waterston J., Seemungal B., Carey J., Bisdorff A., Versino M., Evers S., Newman-Toker D. (2012). Vestibular migraine: Diagnostic criteria. J. Vestib. Res..

[35] Lechner C., Taylor R.L., Todd C., Macdougall H., Yavor R., Halmagyi G.M., Welgampola M.S. (2014). Causes and characteristics of horizontal positional nystagmus. J. Neurol..

[36] Young A.S., Nham B., Bradshaw A.P., Calic Z., Pogson J.M., D’Souza M., Halmagyi G.M., Welgampola M.S. (2021). Clinical, oculographic, and vestibular test characteristics of vestibular migraine. Cephalalgia.

[37] Polensek S.H., Tusa R.J. (2010). Nystagmus during Attacks of Vestibular Migraine: An Aid in Diagnosis. Audiol. Neurotol..

[38] ElSherif M., Reda M.I., Saadallah H., Mourad M. (2020). Eye movements and imaging in vestibular migraine. Acta Otorrinolaringol. Esp..

[39] De Schutter E., Adham Z.O., Kattah J.C. (2019). Central positional vertigo: A clinical-imaging study. Prog. Brain Res..

[40] Carmona S., Martínez C., Zalazar G., Moro M., Batuecas-Caletrio A., Luis L., Gordon C. (2016). The Diagnostic Accuracy of Truncal Ataxia and HINTS as Cardinal Signs for Acute Vestibular Syndrome. Front. Neurol..

